# Urokinase type plasminogen activator receptor (uPAR) as a new therapeutic target in cancer

**Published:** 2016-11-01

**Authors:** Nunzia Montuori, Ada Pesapane, Francesca W Rossi, Valentina Giudice, Amato De Paulis, Carmine Selleri, Pia Ragno

**Affiliations:** 1 Department of Translational Medical Sciences, University Federico II, Naples, Italy; 2 Department of Medicine and Surgery, University of Salerno, Salerno, Italy; 3 Department of Chemistry and Biology, University of Salerno, Salerno, Italy

**Keywords:** uPAR, metastasis, plasminogen activation, monoclonal antibody, small molecules

## Abstract

The urokinase (uPA)-type plasminogen activator receptor (uPAR) is a GPI-anchored receptor that focuses urokinase (uPA) proteolytic activity on the cell surface. uPAR also regulates cell adhesion, migration and proliferation, protects from apoptosis and contributes to epithelial mesenchymal transition (EMT), independently of uPA enzymatic activity. Indeed, uPAR interacts with beta1, beta2 and beta3 integrins, thus regulating their activities. uPAR cross-talks with receptor tyrosine kinases through integrins and regulates cancer cell dormancy, proliferation and angiogenesis. Moreover, uPAR mediates uPA-dependent cell migration and chemotaxis induced by fMet-Leu-Phe (fMLF), through its association with fMLF-receptors (fMLF-Rs). Further, uPAR is an adhesion receptor because it binds vitronectin (VN), a component of provisional extracellular matrix. High uPAR expression predicts for more aggressive disease in several cancer types for its ability to increase invasion and metastasis. In fact, uPAR has been hypothesized to be the link between tumor cell dormancy and proliferation that usually precedes the onset of metastasis. Thus, inhibiting uPAR could be a feasible approach to affect tumor growth and metastasis.

Here, we review the more recent advances in the development of uPAR-targeted anti-cancer therapeutic agents suitable for further optimization or ready for the evaluation in early clinical trials.

## I. INTRODUCTION

The urokinase (uPA)-mediated plasminogen activation system is involved in various pathologic processes, including angiogenesis, inflammation, wound healing, and metastasis [1].

The uPA receptor (uPAR) is anchored to the plasma membrane by a GPI moiety and is formed by three homologous domains (DI, DII, and DIII, from the N-terminus). The uPA-binding site is located in the DI domain, but the full-length molecule is required for an efficient binding [2,3]. uPAR enhances pericellular proteolysis by serving as a docking site to uPA, thus triggering a cascade of proteolytic events that leads to the active degradation of extracellular matrix (ECM) components [4]. The crystal structure of uPAR bound to an antagonist peptide [2] or to the amino-terminal fragment (ATF) of uPA has been solved [3]. uPA is embedded in a central cavity and the large outer receptor surface is available to bind additional ligands and to interact with other cell surface receptors to form a dynamic multiprotein signaling complex [5]. Indeed, despite the lack of a transmembrane domain, uPAR can activate intracellular signaling through lateral interactions with other cell surface receptors, such as integrins, receptor tyrosine kinases, and G-protein–coupled chemotaxis receptors [1]. uPAR ability to regulate integrin activity plays a key role in cell adhesion, migration, proliferation, and survival [1]. Integrin-binding sites have been identified in uPAR domain DII (peptide D2A, residues 130–142) and in uPAR domain DIII (residues 240–248). Soluble D2A abolishes uPAR-alphavbeta3 and uPAR-alpha5beta1 co-immunoprecipitation, indicating that it can bind both of these integrins; in addition, D2A has chemotactic activity that requires alphavbeta3 and activates alphavbeta3 signaling pathways [6]. The specific sequence identified in the uPAR domain DIII binds the alpha5beta1 integrin; substituting a single amino acid in that region (S245A) impairs uPAR binding to the purified integrin [7]. The interaction of alpha5beta1 integrin with uPAR activates integrins; then, through an “outside in” activation process, EGF receptor (EGFR) is recruited to the complex leading to ERKs activation and cell proliferation, thus regulating the shift from tumor cell dormancy to proliferation.

uPAR is also an adhesion receptor; it binds vitronectin (VN), an abundant component of provisional ECM [8]. The X-ray structure of the ternary complex between uPAR, the aminoterminal fragment of uPA (ATF) and the somatomedin B domain of VN (SMB) has been recently determined [9]. There is now evidence that uPAR–VN interaction is entirely mediated by a composite epitope exposed on the DI/DII interface of uPAR (residues R30, W32, S56, R58, I63, S65, S88, R91, R116, and Q114) [10]. uPAR interactions with integrins and VN are positively regulated by uPA [11], and both uPA and VN can induce uPAR-mediated cytoskeletal reorganization and cell migration [12]. uPAR can induce phenotypic changes consistent with hypoxia-induced epithelial–mesenchymal transition (EMT), through a direct binding to VN [8]. Abnormal uPAR levels, as occurring in cancer, may favour EMT through VN binding and RAC-1 activation, thus facilitating tumor invasion and metastasis [9,13].

uPAR interaction with chemotaxis receptors for fMet-Leu-Phe (fMLF-Rs) is required for both uPA- and fMLF-dependent cell migration and occurs through a chemotactic domain located in the DI–DII linker region of uPAR, the SRSRY sequence (amino acids, 88–92) [14]. In fact, a soluble cleaved form of uPAR, lacking in the DI domain and exposing at the N-terminus the SRSRY sequence, is a ligand for fMLF-Rs [15]. The uPAR DI–DII linker region is extremely sensitive to various proteases, including uPA; the proteolytic cleavage removes DI and generates a shorter uPAR form (DIIDIII-uPAR), unable to bind both uPA and VN but, when exposing the SRSRY sequence (aa 88–92) at its N-terminus, still able to interact with fMLF-Rs [1,14]. It has been proposed that the SRSRY sequence is masked in cell-surface uPAR and that uPA binding to uPAR induces a conformational modification causing its exposure and association with members of the fMLF-R family, thus inducing their activation and promoting chemotaxis [16]. We have shown that DIIDIII-uPAR promotes ovarian cancer cell dissemination by driving cell migration and angiogenesis in a protease-independent manner [17].

High uPAR expression predicts for more aggressive disease in several cancer types, for its ability to regulate invasion and metastasis, cancer cell survival and angiogenesis [18]. uPAR has been found to be a key player in regulating the shift between single cell tumor dormancy and proliferation, that usually precedes the onset of metastasis [19]. Indeed, circulating and bone marrow cancer cells express uPAR [20]; simultaneous uPAR and HER2/neu gene amplification on circulating cancer cells has also been described [21]. In gastric cancer, uPAR expression on cancer cells in bone marrow is a prospective predictor of proliferation of these cells and shorter patient survival [22]. Thus, ability of uPAR to coordinate binding and degradation of ECM with cell signaling makes it an attractive therapeutic target in cancer, especially in attempting to prevent/block metastasis.

## II. uPAR INHIBITION BY RNA ANTISENSE METHODOLOGIES

Antisense technologies blocking uPAR expression have evolved from the classic antisense oligodeoxynucleotides methodologies to cell transfection with vectors expressing the antisense transcript complementary to uPAR mRNA. Antisense RNA technology for downregulation of uPAR expression *in vivo* has employed both plasmid and adenovirus constructs. *In vivo*, a reduction of 80% in tibial tumour volumes and total inhibition of pulmonary metastases were observed in mice injected with osteosarcoma cells transfected with an antisense uPAR vector [23]. A human glioblastoma cell line transfected with a cDNA construct corresponding to 300 bp of the human uPAR 5′, after intracerebral injection in nude mice, failed to form tumors [24]. Injection into nude mice, through the tail vein, of H1299 lung cancer cells, infected with a uPAR antisense adenovirus construct, reduced the incidence of lung metastasis by 85% as compared with the control cells [25]. After intracranial injection, a glioblastoma cell line, infected with a bicistronic construct containing antisense sequences of uPAR and uPA in a single adenoviral vector, showed inhibited invasiveness and tumorigenicity; subcutaneous injections of bicistronic antisense constructs into established tumors caused their regression [26].

RNA interference (RNAi) has provided new avenues for the treatment of cancer. RNAi is a RNA-guided regulation of gene expression in which duplexes of 21-nt RNAs, known as short-interfering RNAs (siRNA), pair with a messenger RNA (mRNA) molecule and induce its degradation by the RNA-induced silencing complex (RISC). siRNAs can also be expressed intracellularly, mainly through the use of plasmids containing the RNA pol III promoter; in this method, short hairpin RNAs (shRNA) can function like siRNA duplexes to inhibit gene expression in a sequence-specific manner. siRNAs are believed to be more potent inhibitors of gene expression with less toxicity [27]. A shRNA-based RNAi plasmid system has been employed for the downregulation of uPAR in prostate cancer, resulting in partial reduction of pre-established orthotopic prostate tumor in athymic male nude mice with no observable secondary tumor [28]. Intra-peritoneal injections of a siRNA expressing plasmid targeting both uPAR and uPA in mice with pre-established intracranial gliomas determined tumor regression [29]. In malignant meningiomas, intratumoral injections of the same constructs resulted in regression of pre-established, subcutaneous tumors in mice; the same *in vivo* study also revealed inhibition of intracranial tumor formation [30].

The cellular machinery required for siRNA activity in mammalian cells is the same that physiologically works to regulate the normal mechanisms of gene expression Indeed, key players in the post-transcriptional regulation of gene expression are small non-coding RNAs, termed microRNAs (miRs). MiRs are regulatory single-strand RNAs that typically consist of 20–23 nucleotides in length; they regulate gene expression by pairing with target mRNAs, thus inhibiting their translation and, often, inducing their degradation [31]. We identified three miRs, miR-146a, miR-335 and miR-622, regulating the expression of both uPAR and CXCR4, the receptor of the stroma-derived factor 1 (SDF1) chemokine, in AML cell lines. These miRs directly target the 3′untranslated region of both uPAR- and CXCR4-mRNAs; accordingly, uPAR/CXCR4 expression was reduced by their overexpression in AML cells causing impaired migration, invasion and proliferation of myelomonocytic cells. An inverse relationship between uPAR/CXCR4 expression and miR-146a and miR-335 levels was also found in blasts from AML patients [32]. This observation could be particularly relevant for AML diffusion; indeed, both uPAR and CXCR4 are involved in hematopoietic stem cell (HSC) trafficking [33,34]. In addition, both cell-surface and soluble cleaved uPAR are able to regulate the activity of CXCR4, by a fMLF-R-dependent mechanism [35]. Thus, miR-146a, whose deletion in mouse models leads to myeloproliferative disorders, might represent a useful tool for future therapeutic approaches.

## III. uPAR TARGETING BY MONOCLONAL ANTIBODIES

Recently, a novel uPAR-targeting monoclonal antibody (MoAb), ATN-658, has been identified and developed. That MoAb does not block the binding of uPA or VN to uPAR but inhibits migration and invasion in vitro and demonstrates robust anti-tumor effects in a number of different animal xenograft models of solid tumors. In these models, anti-tumor effects of this humanized MoAb have been observed regardless of tumor histology. Indeed, beside inhibiting metastasis in vivo, as expected for an uPAR-targeting agent, ATN-658 is also able to inhibit tumor proliferation and survival by inhibiting many uPAR-derived signals. In fact, ATN-658 binds the DIII domain of uPAR, close to the C-terminus of the receptor where are located uPAR binding regions for the integrin CD11b (αM), a previously identified uPAR ligand [36].

We characterized a polyclonal antibody targeting the SRSRY sequence of the DI-DII linker region of uPAR; this antibody was able to block uPAR interaction with f-MLFRs and to inhibit uPA- and f-MLF-dependent cell adhesion and migration, as well as uPAR regulation of CXCR4 activity, thus suggesting that this region can represent a suitable target for new monoclonal antibodies in future therapeutic approaches [4,14,35].

## IV. uPAR TARGETING BY SMALL MOLECULES

Using a target structure guided computation docking, 2 compounds, 2-(Pyridin-2-ylamino)-quinolin-8-ol and 2,2′-(methylimino)di (8-quinolinol), able to inhibit ERK activation by destroying uPAR/alpha5beta1integrin association and induce tumor cell dormancy were identified. These two compounds, when applied in vivo, inhibited ERK activity and tumor growth and blocked metastasis in a model of head and neck carcinoma [37].

A small molecule (IPR-456) inhibiting uPAR/uPA interaction was discovered by a virtual screening (VS) approach [38]. IPR-456 and its derivative IPR-803 inhibited uPA binding to uPAR and cell invasion of breast MDA-MB-231 tumor cells, exerting only a little effect on their migration and no effect on their adhesion [39]. Two additional derivatives of IPR456 showed an inhibitory effect on cell invasion, migration and adhesion of non-small cell lung cancer (NSCLC) cell lines. However, the effects on invasion of these active compounds were consistent with their capability to inhibit uPA and MMP proteolytic activity [40]. In a subsequent study, IPR-803, orally administered to female mice inoculated with highly malignant TMD-MDA-MB-231 cells in their mammary fat pads, impaired metastasis formation to the lungs and demonstrated a promising activity as a template for the development of anti-metastasis drugs [41].

We used a structure-based virtual screening (SB-VS) approach to search for small molecules targeting the uPAR-binding site for VN. Indeed, uPAR promotes metastasis by engaging VN and activating cell signaling pathways [13]. Two compounds, 6 and 37, selectively inhibited uPAR-dependent cell adhesion to VN and the resulting signal transduction. Both compounds targeted S88 and R91, key residues for uPAR binding to VN but also for uPAR interaction with the f-MLFRs, thus impairing cell migration and in vitro ECM invasion of several cancer cell types and representing new promising leads for pharmaceuticals in cancer [42].

uPAR interactions with VN are positively regulated by uPA [11]; thus IPR-803 allosterically inhibited also VN binding to uPAR, explaining its strong anti-metastatic activity [43]

## V. uPAR INHIBITION BY SYNTHETIC PEPTIDES

Peptide technologies applied to uPAR have employed both the classic search for antagonists of uPA binding to the receptor and the search for inhibitors of uPAR interaction with membrane partners.

Recently, a decapeptide antagonist of uPA binding to uPAR was introduced. In the MDA-MB-231 breast cancer cell line, this antagonist peptide caused p38 activation and low ERK activation, down-regulation of Bcl-2 and up-regulation of Bim without Bax modulation, thus exerting a pro-apoptotic effect [44].

A crucial uPAR signaling region is the protease sensitive region linking DI and DII domains; its minimal active 88Ser-Arg-Ser-Arg-Tyr92 sequence is able to trigger cell migration and angiogenesis in vitro and in vivo, even in the form of synthetic linear Ser-Arg-Ser-Arg-Tyr peptide (SRSRY). To inhibit this uPAR sequence, a family of penta-peptides carrying the S90E substitution in the uPAR_88–92_ sequence was synthetized. The peptide pERERY-NH2 inhibited uPAR/fMLF-R interaction, thus blocking migration of various tumor cell lines [45]. Subsequently, new tetra-peptides having the general formula Ac-Arg-Glu-Arg-X-NH2 (X = Phe, Tyr, Trp) were synthesized, and the peptide RERF also potently prevented in vitro and in vivo cell migration and invasion [46]. Recently, it was found that RERF also behaved as an antiangiogenic agent by inhibiting in vitro and in vivo responses either to uPAR_88–92_ or to VEGF. RERF prevented the recruitment of αvβ3 integrin at the focal adhesions in endothelial cells exposed to VEGF, by forcing αvβ3 in an inactive state either directly or indirectly, through fMLF-Rs [47]. Thus, N-acetylated and C-amidated peptide analogues with optimized properties for therapeutic applications were synthesized. Ac-L-Arg-Aib-L-Arg-D-Cα(Me)Phe-NH2, named UPARANT, in vivo, prevented VEGF-dependent angiogenesis. Both excellent stability and potency make UPARANT as a promising new therapeutic agent for the control of diseases with excessive angiogenesis, such as cancer and inflammation [48].

Recently, cyclization of the Ser-Arg-Ser-Arg-Tyr peptide was attempted to generate a new, more stable peptide that could regulate uPAR_88–92_-dependent functions. Cyclized [SRSRY] peptide competed with fMLF for binding to fMLF-R type 1 (FPR1) and inhibited cell migration in a dose-dependent manner. In vivo, cyclized [SRSRY] peptide reduced intestinal inflammation impairing recruitment of inflammatory monocytes to the inflamed tissue and exerted an anti-metastatic effect by reducing in vivo vascular infiltration by chondrosarcoma cells [49].

## VI. CONCLUSION

Despite an abundance of literature demonstrating the importance of uPAR in the progression of most solid cancers, including breast, colon, prostate, pancreatic, ovarian, lung, and brain as well as several hematologic malignancies such as acute leukemia and myeloma, no uPAR targeting therapeutic agents have been developed or evaluated in cancer clinical trials to date. A number of antibodies that directly inhibit the binding of uPA to uPAR have been proposed and tested in pre-clinical studies but most of these have only demonstrated modest antitumor activity and were therefore never advanced into the clinic. Recently, the humanized MoAb, ATN-658, inhibiting both metastasis and tumor proliferation in vivo, has emerged as a new promising tool for starting early clinical trials.

Among small molecules, IPR-803 is a promising template for the development of anti-metastasis drugs, for its *in vivo* activity. Small molecules are advantageous in respect to biological agents; indeed, they are orally available, cell permeable and highly specific [50]. uPAR-targeting small molecules are expected to act only in cells with high uPAR expression, a property almost exclusive to cancer cells; thus, new therapies based on these compounds are expected to be cancer cell specific and minimally toxic. These treatments, rather than be directly cytotoxic, will block disseminated metastatic cells, preventing/treating overt metastasis.

The cyclic [SRSRY] and UPARANT peptides are also very recent promising tools for future therapies, given their selective ability to target in vivo tumor angiogenesis and vascular infiltration.

## Figures and Tables

**Figure 1:**
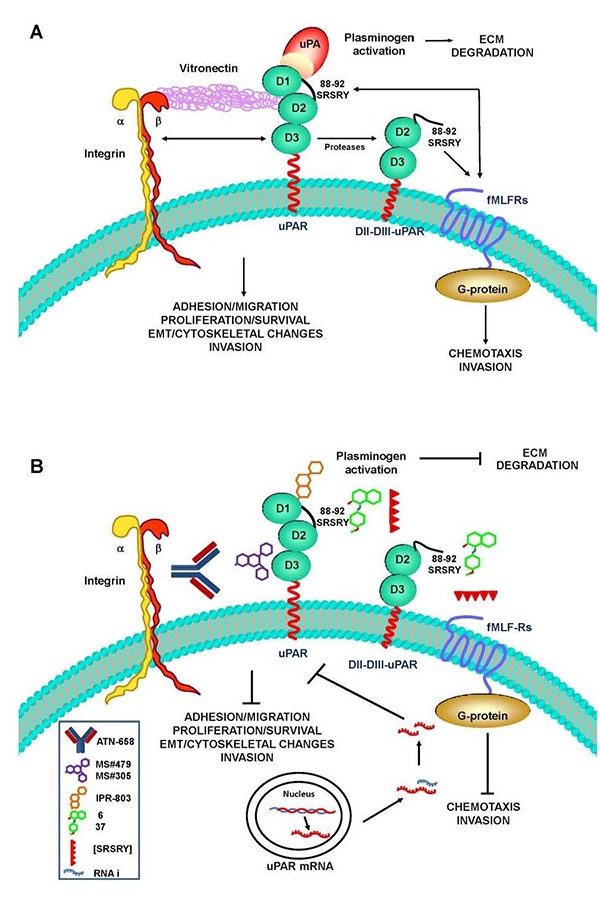
The latest approaches to uPAR targeting in cancer. PANEL A. uPAR focuses uPA proteolytic activity on the cell surface leading to the active ECM degradation. uPAR also regulates cell adhesion, migration and proliferation, protects from apoptosis, induces cytoskeletal rearrangements and contributes to epithelial-mesenchymal transition (EMT), independently from uPA enzymatic activity, through its interaction with betal, beta2 and beta3 integrins. uPAR is also an adhesion receptor; it binds vitronectin (VN). Moreover, uPAR mediates chemotaxis and invasion through its functional association with fMLF-receptors (fMLF-Rs). PANEL B. Humanized MoAb ATN-658 and MS#479/MS#305 small molecules target uPAR interaction with integrins, thus inhibiting cell proliferation and inducing apoptosis. IPR-803 small molecule targets uPA binding to uPAR, thus inhibiting ECM degradation and uPA-mediated signals. Compounds 6 and 37 as well as the cyclic [SRSRY] peptide target uPAR interaction with fMLF-Rs, thus inhibiting cell migration and invasion as well as angiogenesis. RNA interference (RNAi) induces downregulation of uPAR mRNA and protein resulting in inhibition of all receptor functions.
